# Association of Air Pollution and Heat Exposure With Preterm Birth, Low Birth Weight, and Stillbirth in the US: A Systematic Review

**DOI:** 10.1001/jamanetworkopen.2020.8243

**Published:** 2020-06-18

**Authors:** Bruce Bekkar, Susan Pacheco, Rupa Basu, Nathaniel DeNicola

**Affiliations:** 1 Retired from Southern California Permanente Medical Group, San Diego; 2 The University of Texas McGovern Medical School, Houston; 3 California Office of Environmental Health Hazard Assessment, Air and Climate Epidemiology Section, Oakland; 4 Department of Environmental Health Sciences, University of California Berkeley School of Public Health, Berkeley; 5 George Washington University School of Medicine and Health Sciences, Washington, DC

## Abstract

**Importance:**

Knowledge of whether serious adverse pregnancy outcomes are associated with increasingly widespread effects of climate change in the US would be crucial for the obstetrical medical community and for women and families across the country.

**Objective:**

To investigate prenatal exposure to fine particulate matter (PM_2.5_), ozone, and heat, and the association of these factors with preterm birth, low birth weight, and stillbirth.

**Evidence Review:**

This systematic review involved a comprehensive search for primary literature in Cochrane Library, Cochrane Collaboration Registry of Controlled Trials, PubMed, ClinicalTrials.gov website, and MEDLINE. Qualifying primary research studies included human participants in US populations that were published in English between January 1, 2007, and April 30, 2019. Included articles analyzed the associations between air pollutants or heat and obstetrical outcomes. Comparative observational cohort studies and cross-sectional studies with comparators were included, without minimum sample size. Additional articles found through reference review were also considered. Articles analyzing other obstetrical outcomes, non-US populations, and reviews were excluded. Two reviewers independently determined study eligibility. The Arskey and O’Malley scoping review framework was used. Data extraction was performed according to the Preferred Reporting Items for Systematic Reviews and Meta-analyses (PRISMA) reporting guideline.

**Findings:**

Of the 1851 articles identified, 68 met the inclusion criteria. Overall, 32 798 152 births were analyzed, with a mean (SD) of 565 485 (783 278) births per study. A total of 57 studies (48 of 58 [84%] on air pollutants; 9 of 10 [90%] on heat) showed a significant association of air pollutant and heat exposure with birth outcomes. Positive associations were found across all US geographic regions. Exposure to PM_2.5_ or ozone was associated with increased risk of preterm birth in 19 of 24 studies (79%) and low birth weight in 25 of 29 studies (86%). The subpopulations at highest risk were persons with asthma and minority groups, especially black mothers. Accurate comparisons of risk were limited by differences in study design, exposure measurement, population demographics, and seasonality.

**Conclusions and Relevance:**

This review suggests that increasingly common environmental exposures exacerbated by climate change are significantly associated with serious adverse pregnancy outcomes across the US.

## Key Points

**Question:**

Are increases in air pollutant or heat exposure related to climate change associated with adverse pregnancy outcomes, such as preterm birth, low birth weight, and stillbirth, in the US?

**Findings:**

In this systematic review of 57 of 68 studies including a total of 32 798 152 births, there was a statistically significant association between heat, ozone, or fine particulate matter and adverse pregnancy outcomes. Heterogeneous studies from across the US revealed positive findings in each analysis of exposure and outcome.

**Meaning:**

The findings suggest that exacerbation of air pollution and heat exposure related to climate change may be significantly associated with risk to pregnancy outcomes in the US.

## Teaser

This systematic review summarizes the association of climate change, including heat, ozone, and fine particulate matter, with adverse pregnancy outcomes, such as preterm birth, low birth weight, and stillbirth, in the US.

## Introduction

The current climate crisis, also known as climate change or global warming, has been widely recognized as an environmental emergency that threatens many critical resources and protections including sustainable food and water supplies, natural disaster preparedness, and US national security.^1,2,3^ However, as the World Health Organization^4^ and The Lancet Countdown^5^ have identified, one of the greatest consequences of climate change is its association with human health.

Specific to women’s health, the American College of Obstetricians and Gynecologists position statement^6^ recognizes that “climate change is an urgent women’s health concern as well as a major public health challenge.”^6^ The associations of climate change with women’s health have been further outlined^7^ to include a wide range of undesirable outcomes, such as worsening of cardiac disease, respiratory disease, and mental health, and exposure to an increasing number of infectious diseases.

These adverse health effects are most consequential to at-risk populations, which include a high number of pregnant women and developing fetuses.^8,9^ The obstetrical literature^10,11^ has included numerous observational studies demonstrating an association between air pollution and heat and increased risk of adverse birth outcomes. Two components of air pollution that are exacerbated by climate change and continued use of fossil fuels are fine particulate matter less than 2.5 μm in diameter (PM_2.5_) and ozone.^12,13^

In this review, we assessed the associations between exposure to PM_2.5_, ozone, and heat and preterm birth, low birth weight, and stillbirth. Although these associations have largely been studied in a global setting,^14,15,16,17^ we focused specifically on the US population, in which these exposures are increasingly common.

## Methods

### Scope of Review

For this systematic review, we evaluated evidence of the association between air pollution and heat on the adverse obstetrical outcomes of preterm birth, low birth weight, and stillbirth. The Arskey O’Malley methodologic framework for a scoping review was used.^18,19^ This study followed the Preferred Reporting Items for Systematic Reviews and Meta-analyses (PRISMA) reporting guideline.

### Research Questions

The following specific key questions were addressed in this review. Is prenatal exposure to PM_2.5_ or ozone associated with increased risk of preterm birth? Is prenatal exposure to PM_2.5_ or ozone associated with increased risk of low birth weight? Is prenatal exposure to PM_2.5_ or ozone associated with increased risk of stillbirth? Is prenatal exposure to heat associated with increased risk of preterm birth? Is prenatal exposure to heat associated with increased risk of low birth weight? Is prenatal exposure to heat associated with increased risk of stillbirth?

### Eligibility Criteria

Studies published from January 1, 2007, to April 30, 2019, on US populations were deemed eligible. Studies that included either PM_2.5_ or ozone with or without other criteria pollutants or heat and the adverse obstetrical outcomes of interest were included.

Detailed study inclusion and exclusion criteria for the systematic review are given in the Figure and eTable 1 in the Supplement. Studies were included and assessed for appropriateness if they satisfied the PICOTS elements: population, exposure, comparator, outcome, time, and study location.

**Figure.**
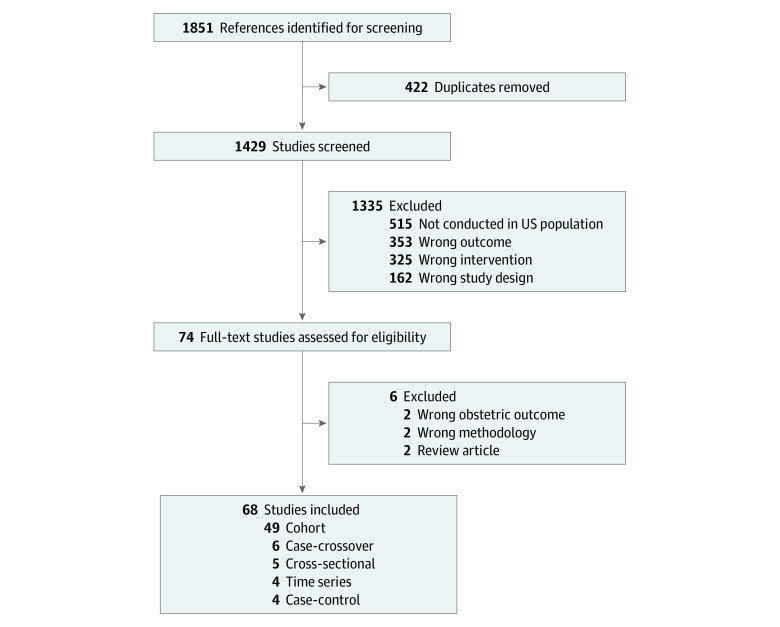
PRISMA Flow Diagram

### Study Selection

Comprehensive literature searches were performed using the ClinicalTrials.gov website, PubMed, MEDLINE, Cochrane Library, and Cochrane Collaboration Registry of Controlled Trials. Medical Subject Headings (MeSH) terms are given in the Supplement. Two of us (B.B. and N.D.) evaluated studies pertinent to each research question. In phase 1, articles were selected by study type and primary outcome. Comparative observational cohort studies and cross-sectional studies with comparators were included. There was no requirement for minimum sample size for inclusion. In phase 2, articles were selected by type of obstetrical outcome. Studies that included secondary obstetrical outcomes in addition to preterm birth, low birth weight, and stillbirth were included; however, studies with other primary obstetrical outcomes, such as preeclampsia and asthma exacerbation, were excluded. Review articles were excluded, but additional articles found through individual review of references were screened for eligibility.

### Charting Data, Collating, Summarizing, and Reporting Results

With use of the Arskey O’Malley methodologic framework, study selection was focused post hoc to the exposures and outcomes specified. Race/ethnicity was identified in accordance with the studies reviewed and was analyzed to determine whether one race/ethnicity appeared to have a stronger association than others present in these categories. Results were organized into summary of evidence tables and presented qualitatively to describe the degree of heterogeneity in study designs, types of exposures, outcome measurements, and settings. Because of heterogeneity of environmental exposures among the studies and the variable manner of data reporting, quantitative estimates of pooled effects were not generated.

### Statistical Analysis

A pooled analysis or meta-analysis was not performed because of the heterogeneity of the study populations across geographic locations and the heterogeneity of exposures (ie, PM_2.5_ from vehicle traffic and wildfires). The review presented the primary findings in summary of evidence tables for each key question and tabulated the preponderance of evidence that found significant associations (ie, 19 of 24 studies on preterm birth and air pollution found a significant association). The overall number of births were included in the review, and the Table lists the number of births reviewed for each key question, and the mean (SD) births among these studies. This was presented to highlight the large populations being studied that provided credence to the significant findings. The degree of risk was identified for significant associations as a range with median. These data were tabulated and calculated with an Excel spreadsheet (Microsoft).

**Table.** Summary of Evidence Key Questions 1 Through 6

Exposure and outcome	Studies finding an association, No./total No.	Births/study, mean (SD)	Total births in millions	Increased risk, median (range), %^a^	Studies finding racial disparity, No./total No.	Notable findings^b^
Air pollution						
Preterm birth	19/24	318 960 (393 272)	7.3	11.5 (2.0-19.0)^c^	10/19	Preterm birth risk increased 52% for asthmatic mothers
Low birth weight	25/29	661 205 (878 074)	18.5	10.8 (2.0-36.0)^c^	13/25	Low birth weight risk increased 3% for each 5-km proximity to a solid waste plant
Stillbirth	4/5	1 020 975 (1 176 174)	5.1	14.5 (6.0-23.0)^c^	1/4	Stillbirth risk increased 42% with high third-trimester exposure
Heat						
Preterm birth	4/5	192 625 (207 995)	0.8	15.8 (9.0-22.0)^d^	2/4	Preterm birth risk increased 11.6% per 5.6 °C increase
Low birth weight	3/3	902 277 (985 803)	2.7	31.0 (13.0-49.0)^d^	1/3	Term birth weight decreased 16 g per IQR temperature increase
Stillbirth	2/2	115 943 (115 933)	0.2	NA^e^	2/2	Stillbirth risk increased 6% per 1 °C increase the week before delivery during the warm season

Abbreviations: IQR, interquartile range; NA, not applicable.

^a^ Risk presented as range from significant studies. The median is calculated from the range; a pooled analysis was not performed. For consistency, the whole pregnancy exposure was presented where possible.

^b^ Single study unless specified.

^c^ For whole pregnancy PM_2.5_ exposure.

^d^ For whole pregnancy heat exposure.

^e^ The only 2 studies on heat and stillbirth did not provide comparable outcomes that could be combined into a range with a median.

### Results

A total of 1851 articles matched our search terms, of which 68 articles met our study criteria: 58 (85%) on air pollutants and 10 (15%) on heat (Figure). There were 49 (72%) cohort studies, 6 (9%) case-crossover studies, 5 (7%) cross-sectional studies, 4 (6%) time series studies, and 4 (6%) case-control studies. A total of 32 798 152 births were analyzed, with a mean (SD) of 565 485 (738 278) births per study and a range of 670 to 3 012 270 births per study.

Regarding exposure to PM_2.5_ and ozone, there were 24 (41%) studies on preterm birth, 29 (50%) on low birth weight, and 5 (9%) on stillbirth. Of the 58 studies addressing air pollution, 56 (96%) included PM_2.5_, 23 (40%) included ozone, and 21 (36%) analyzed both. Of these, 48 studies (84%) found a significant association between exposure to air pollutants and adverse birth outcomes (Table).

We found 10 studies examining the association between heat exposure and obstetrical outcomes: 5 (50%) on preterm birth, 3 (30%) on low birth weight, and 2 (20%) on stillbirth. Nine of the 10 articles (90%) found a significant association between exposure to heat during pregnancy and adverse birth outcomes (Table).

There were 24 included studies evaluating the association of maternal exposure to PM_2.5_ and/or ozone with preterm birth (eTable 2 in the Supplement); 19 studies (79%) found an increased risk. Each of these studies included PM_2.5_; 7 (37%) also analyzed ozone.

Of the 11 studies analyzing PM_2.5_ whole-pregnancy exposure, the risk increased by a median of 11.5% (range, 2%-19%). Six of these reports (54%) measured associations with preterm birth using a whole-pregnancy median of 3.9 μg/m^3^ (interquartile range [IQR], 1.35-6.45 μg/m^3^) of PM_2.5_ exposure. For example, in a study of traffic-generated PM_2.5_ in Los Angeles and Orange County, California, Wu et al^20^ found that preterm birth overall increased 3% (95% CI, 1%-6%), deliveries less than 35 weeks increased 7% (95% CI, 3%-12%), and deliveries less than 30 weeks increased 18% (95% CI, 10%-26%) per IQR of 1.35 μg/m^3^.

Of the 4 studies analyzing ozone whole-pregnancy exposure, 2 (50%) found an increased risk from 3% to 9.6%^21,22^; each measured the association by IQR, from 7.1 to 11.53 parts per billion (ppb). Ha et al^21^ found an overall 3% increased risk of delivery before 37 weeks and 13% increased risk for deliveries before 32 weeks per IQR of 7.1 ppb. In a report on 34 705 singleton births and first-trimester ozone exposure in Pennsylvania, Lee et al^23^ found a 23% increase in risk per IQR of 16.8 ppb.

Ten studies reported the association of racial/ethnic disparities with increased risk of preterm birth among mothers in minority groups; 8 of the studies noted higher risk for black mothers, which was the most consistent finding among the subgroups. One study analyzing subgroups revealed a higher risk for preterm birth among patients with asthma.^24^ Other subgroups identified in 2 studies each included younger and older mothers, those with less educational level, and those with government insurance or lacking early prenatal care.

Five studies showed no association between PM_2.5_ and preterm birth, measuring exposures during the whole pregnancy, by trimester, or by month of birth.^25,26,27,28,29^ Salihu et al^29^ found no significant association with preterm birth and above the median 11.28 μg/m^3^ PM_2.5_ exposure overall in a retrospective cohort in Florida but showed an 8% increased risk of preterm birth in association with 3-way interaction of PM_2.5_, fine particulate matter less than 10.0 μm in diameter, and coarse particulates. Another study^28^ used a nationally representative sample of higher-risk births and found no association of ozone exposure and a 3% odds per parts per million reduction in preterm birth rates with PM_2.5_.

There were 29 included studies evaluating the association of maternal exposure to PM_2.5_ and/or ozone with low birth weight (eTable 3 in the Supplement); 25 studies found an increased risk. All studies except 1 included PM_2.5_; 11 analyzed ozone, 10 of them in combination with PM_2.5_.

Eight studies examining whole-pregnancy exposure to PM_2.5_ found a 2% to 36% increased risk of low birth weight. One study from Florida^30^ reported a 3% increased risk of low birth weight for each 5 km nearer residential proximity to a solid waste plant emitting PM_2.5_. Five of the 8 studies (62%) of whole-pregnancy exposure showing increased risk of low birth weight analyzed the association of IQR increases in PM_2.5_, which ranged from 2.0 to 6.9 μg/m^3^. In Massachusetts and Connecticut, Hyder et al^10^ found an 8% increased risk per IQR increases of 2.41.

Three studies^31,32,33^ found that whole-pregnancy exposure to ozone was associated with a significant increase in risk of low birth weight; 2 of them^32,33^ noted a 6% to 13% increased risk per IQR (7.4-11.5 ppb). Among 74 416 live births in Orange County, California, Laurent et al^33^ found a 13% (95% CI, 2%-25%) greater risk of low birth weight per IQR increase (11.5 ppb) in ozone.

Thirteen studies reported the association of racial/ethnic disparities with increased risk of low birth weight among mothers in minority groups; 12 were cohort studies, and 1 was a case-control analysis. As with preterm birth and air pollutants, the most frequently noted high-risk subpopulation was black mothers in 10 of 13 studies (77%); 4 (31%) noted higher risks among Asians, and 3 (23%) among Hispanics. Three other studies^32,34,35^ noted an association of lower socioeconomic status or living in older homes, near roadways, or in urban cores to with increased risk.

Three cohort studies and 1 case-control study showed no associations with low birth weight,^36^ raw birth weight, gestational age *Z* scores,^37,38^ or small for gestational age.^23^ All studies analyzed PM_2.5_, and 2 added ozone.^23,36^ One study of PM_2.5_ exposure did find a significant association with low birth weights in 1 subpopulation: –0.42 raw birth weight, gestational age *Z* score (95% CI, –0.79 to –0.06) per IQR for male infants of obese mothers.^38^

There were 5 included studies evaluating the association of maternal exposure to PM_2.5_ and/or ozone with stillbirth (eTable 4 in the Supplement); 4 found an increased risk.^39,40,41,42^

For PM_2.5_, a study by DeFranco et al^41^ of more than 350 000 births in Ohio noted an increased risk (42%; 95% CI, 6%-91%)of stillbirth associated with high exposure during the third trimester. The studies of prenatal ozone exposure either during the whole pregnancy, the third trimester, or the week before delivery identified a range of increased risk of stillbirth from 3% to 39%.^40,42^ A cause-specific analysis by Ebisu et al^39^ found the highest risk of 23% (95% CI, 6%-44%) for fetal growth–related stillbirths in association with PM_2.5_ whole-pregnancy exposure per IQR increase (7.23 μg/m^3^).

Two cohort studies noted subpopulations at higher risk: mothers with asthma^40^ and Hispanic mothers.^42^ In an analysis of 12 clinical sites across the US by Mendola et al,^40^ mothers with asthma were found to be especially susceptible to stillbirth if exposed to whole-pregnancy elevated PM_2.5_. A report of ozone exposure^42^ using statewide data from California found Hispanic mothers to be at higher risk from whole-pregnancy 10-ppb increases at mean (SD) levels of 48.48 (12.48) ppb.

One study from New Jersey^43^ noted no association of stillbirth per trimester or whole pregnancy with prenatal PM_2.5_ exposures, although it did find a significant association of stillbirth with prenatal exposure to carbon monoxide, nitrogen dioxide, and sulfur dioxide; ozone was not included. Of note, an analysis by Green et al^42^ of ozone and PM_2.5_ exposure in California did not detect a significant association of stillbirth per 10 μg/m^3^ of PM_2.5_ with whole-pregnancy exposure.

There were 5 included studies evaluating the association of maternal exposure to heat with preterm birth (eTable 5 in the Supplement). A total of 4 (80%) found an increased risk.

Temperature was reported as either weekly mean apparent temperature^44,45,46^ or, in 1 analysis, weekly mean extreme temperature, with extreme heat as the 90% and extreme cold as the 10% of ambient weekly temperature.^47^ These 4 studies identified a range of increased risk of preterm birth from 8.6% to 21.0%.

Three studies^44,45,46^ examining large numbers of preterm births (range, 14 466-58 681 births) in California noted an increased risk of preterm birth for each 5.6 °C-increase in temperature, as did another study covering 12 clinical sites across the US for 2.8 °C increase.^47^

Two reports from California^45,46^ found an association of racial/ethnic disparity and heat exposure with an increasing risk of preterm birth; higher risk was found among black mothers. Increased risk of preterm birth was also found for Asian mothers and younger mothers in Basu et al.^46^

One cross-sectional analysis did not identify a significant association with preterm birth and heat exposure. Kloog et al’s^48^ satellite-based spatial modeling technique in Massachusetts found no association with preterm birth (1.04; 95% CI, 0.96-1.13) and a small reduction in gestational age at delivery (–0.26%; 95% CI, –0.28% to –0.25%) per 2.8 °C whole-pregnancy mean ambient temperature increase. Of note, standard monitoring data with similar elevated temperatures showed an association with preterm birth (1.02; 95% CI, 1.00-1.05).

There were 3 included studies evaluating the association of maternal exposure to heat with low birth weight (eTable 6 in the Supplement); all found an increased risk.^48,49,50^ Heat was reported as a range of mean apparent temperature^48,49^ or as greater than 95% for a specific location and period to account for acclimatization.^50^

Each study identified a specific risk in the third trimester; Basu et al^49^ and Ha et al^50^ found increased risk of low birth weight at term. The analysis by Basu et al^49^ of 2 076 230 live births in California found a 15.8% (95% CI, 5.0%-27.6%) increased risk of low birth weight per 5.6 °C exposure in the third trimester and 13% (95% CI, 4.1%-22.7%) for whole pregnancy. The analysis of Ha et al^50^ of 12 clinical sites across the US showed that ambient local temperature greater than 95% was associated with a relative risk of low birth weight of 1.31 (95% CI, 1.15-1.50) for third-trimester exposure and 2.49 (95% CI, 2.20-2.83) for the whole pregnancy.

In the analysis by Kloog et al^48^of 459 019 live births in Massachusetts, there was a 16.7-g (3.7-29.7–g) weight reduction at term per IQR increased temperature (8.4 °C) in the third trimester; low birth weight was not significantly increased per 2.8 °C increase in whole pregnancy temperature.

 Ha et al^50^ found that extreme cold (<5%) exposures during second and third trimesters and the whole pregnancy were also significantly associated with low birth weight at term.

There were 2 studies evaluating the association of maternal exposure to heat with low birth weight, and both found an increased risk (eTable 7 in the Supplement).^51,52^ Heat was reported per 5.6°C of warm season mean apparent temperature,^52^ and Ha et al^51^ measured associations of both mean temperature in the week before delivery and extreme temperature, defined as either greater than 90% or less than 10% of ambient temperature exposure during the whole pregnancy.

In a case-crossover analysis in California, Basu et al^52^ found an increased risk of 10.4% per 5.6 °C in mean ambient temperature (cumulative average of lags, 2-6 days). The nationwide study by Ha et al^51^ reported an increase of 6% per 1.0 °C in the week before delivery during the warm season; in both studies the authors accommodated for an estimated 1 week from exposure to fetal expulsion. Both studies^51,52^ noted higher risks for younger or older mothers and minority racial/ethnic groups; one each indicated poorer outcomes for black or Hispanic mothers.

## Discussion

Studies across diverse US populations were identified that reported an association of PM_2.5_, ozone, and heat exposure with the adverse obstetrical outcomes of preterm birth, low birth weight at term, and stillbirth. More than 32 million births were analyzed, with a mean (SD) of 565 485 (783 278) births per study. In each analysis of climate change–related exposure and adverse obstetrical outcome, most of the studies found a statistically significant increased risk (Table). The highest number of studies (eTables 2-7 in the Supplement) were found for risk of preterm birth (29 studies) and low birth weight (32 studies), whereas limited studies were identified for stillbirth (7 studies) because of the lack of available data for health studies.

Our review contributes the largest number of recent studies (2007-2019) focusing solely on US populations and is the first, to our knowledge, to combine the increasingly common exposures of air pollutants and heat associated with a series of adverse obstetrical outcomes. Our findings are consistent with other review articles that were not included in our analysis (all included non-US participants). Reviews that examined PM_2.5_ found consistently positive association with preterm birth and low birth weight or continuous birth weight,^16,17^ and 1 systematic review and meta-analysis on stillbirth risk showed elevated effect estimates for both PM_2.5_ and ozone, although they did not achieve significance.^53^ Five reviews that focused on heat exposure found an association with preterm birth in most studies,^14,15,54,55,56^ as did 4 that analyzed low birth weight^14,15,54,56^ and 2 analyzing stillbirth risk.^14,15^

The adverse obstetrical outcomes examined in this study are known to be complex, heterogeneous, and multifactorial in origin; several animal studies suggested that both air pollutant and heat exposure may contribute to adverse obstetrical outcomes.^57,58,59,60^ Regarding preterm birth, mechanisms that implicate toxic fine particulates include maternal hematologic transport of inhaled noxious chemicals, the triggering of systemic inflammation, or alterations in function of the autonomic nervous system.^61,62,63^ Low birth weight may be associated with air pollutants by direct toxic effects from fetal exposure, altered maternal cardiac or pulmonary function, systemic inflammation from oxidative stress, placental inflammation, altered placental gene expression, or changes in blood viscosity; multiple effects may operate simultaneously.^27,64,65,66,67,68^ Mechanisms for the association of air pollutants with stillbirth may involve alterations in oxygen transport, DNA damage, or placental injury.^69,70,71,72^ The cause-specific analysis by Ebisu et al^39^ of stillbirths reinforces the apparent association of injury to the fetal-placental unit with air pollutant exposure compared with other possible causes.

Heat exposure may contribute to prematurity through labor instigation from dehydration (via prostaglandin or oxytocin release), from altered blood viscosity, and/or by leading to inefficient thermoregulation^60,73,74^; it may also trigger preterm premature rupture of membranes and thus preterm birth during the warm season.^75^ Likewise, heat exposure may impair fetal growth by reducing uterine blood flow and altering placental-fetal exchange.^74,76,77^ Mechanisms associated with elevated temperatures and stillbirth include the initiation of premature labor (as noted above), lowering amniotic fluid volume, damaging the placenta,^78^ or causing abruption.^79^

Biologic plausibility is further supported by other recent studies not included in this review. The study by Casey et al^80^ of preterm birth rates in California before and after coal power plant closures showed a 27% reduction during the 10-year period after closure. Currie et al^81^ found that among 1.1 million live births in Pennsylvania, the risk of low birth weight was higher within 3 km of a fracking site compared with the background risk and increased by 25% within 1 km of a site.

This review revealed a disproportionate effect on populations defined as pregnant women with certain medical conditions or specific race/ethnicities. Women with asthma may be particularly susceptible to adverse outcomes, such as preterm birth and stillbirth, in association with PM_2.5_ exposure during gestation.^24,40^ Among racial/ethnic groups, our findings suggest that black mothers are at greater risk for preterm birth and low birth weight. Social determinants of health, including residence in urban areas with higher exposure to air pollutants and long-term high levels of stress, are known to contribute to adverse obstetrical outcomes.^82^ A recent study^83^ from California suggested that PM_2.5_ exposure alone was associated with an equivalent amount of the racial disparity (black vs white) in preterm birth rates as did other demographic and social factors. Our research suggests that these environmental exposures further exacerbate that background risk and could be included among these social determinants.

Regarding both air pollutant and heat exposure, associations with adverse birth outcomes were found across the continental US. For example, studies on air pollution and low birth weight found an association in 19 states in the Northeast (10), Southeast (5), Midwest (2), Mountain (1), and West (1) regions. California, known for both high temperatures and unhealthy particulate and ozone levels,^84^ was included in the greatest number of studies showing a positive association (13), followed by Massachusetts (6), Georgia (5), and Florida (4). The exposures are complex; even within 1 state, the weather patterns, geography, and urbanization may create zones with widely different pollution risks, as shown by Tu et al^31^ in Georgia.

Future research is needed to further identify at-risk populations, high-exposure geographic areas, and effects of seasonality. This ongoing research may be enhanced by improved geographic information systems that can be mapped onto existing US public health databanks such as the Nationwide Inpatient Sample and Kids’ Inpatient Database.^85,86^

### Strengths and Limitations

Strengths of the study include the considerable sample size and the wide geographic range that includes every region of the US domestic population. Although other reviews have included global analysis, our focus on the US population makes the findings particularly relevant to pregnant women and health care clinicians in the US. Also, in research examining diffuse exposures, such as air pollution and heat, in which pooled analysis across studies is often not feasible, there is merit to tabulating the overall preponderance of observations from varying studies examining the same outcomes.

This study has limitations. First, this review covers only observational studies with heterogeneous sources of air pollution and heat exposure as well as diverse methods of measurement. For air pollutant studies, results can vary based on exposure measurement methods,^10,87^ locations and sources analyzed,^88^ and population demographics.^35^ For heat exposures, results may also be affected by demographics,^52^ acclimatization, and seasonal effects.^46,50^ For both air pollution and heat exposure, different study designs may complicate direct comparison of the data even within a single study.^50^ In addition, the number of studies on stillbirth is limited.

## Conclusions

This review suggests that increasingly common environmental exposures exacerbated by climate change are significantly associated with serious adverse pregnancy outcomes across the US. It appears that the medical community at large and women’s health clinicians in particular should take note of the emerging data and become facile in both communicating these risks with patients and integrating them into plans for care. Moreover, physicians can adopt a more active role as patient advocates to educate elected officials entrusted with public policy and insist on effective action to stop the climate crisis.

## Notes

Supplement. **eTable 1.** PRISMA (Preferred Reporting Items for Systematic Reviews and Meta-Analyses) Table and MESH Terms Search **eTable 2.** Air Pollution and Preterm Birth **eTable 3.** Air Pollution and Low Birth Weight **eTable 4.** Air Pollution and Stillbirth **eTable 5.** Heat and Preterm Birth **eTable 6.** Heat and Low Birth Weight **eTable 7.** Heat and Stillbirth
