# Error in Results Section

**DOI:** 10.1001/jamanetworkopen.2020.14510

**Published:** 2020-07-07

In the Original Investigation, “Association of Air Pollution and Heat Exposure With Preterm Birth, Low Birth Weight, and Stillbirth in the US: A Systematic Review,”^1^ published on June 18, 2020, in paragraph 28 of the Results section, there were 2 studies evaluating the association of maternal exposure to heat with stillbirth, not low birth weight. This article has been corrected.
