# Supplementary material for: Association of Air Pollution and Heat Exposure With Preterm Birth, Low Birth Weight, and Stillbirth in the US: A Systematic Review
Source: JAMA Netw Open. 2020 Jun 18;3(6):e208243. doi: 10.1001/jamanetworkopen.2020.8243 (PMC7303808; doi:10.1001/jamanetworkopen.2020.8243)
Supplement: Supplement. — eTable 1. PRISMA (Preferred Reporting Items for Systematic Reviews and Meta-Analyses) Table and MESH Terms Search eTable 2. Air Pollution and Preterm Birth eTable 3. Air Pollution and Low Birth Weight eTable 4. Air Pollution and Stillbirth eTable 5. Heat and Preterm Birth eTable 6. Heat and Low Birth Weight eTable 7. Heat and Stillbirth [file jamanetwopen-3-e208243-s001.pdf]

Supplementary Online Content

Bekkar B, Pacheco S, Basu R, DeNicola N. Association of air pollution and heat exposure with preterm birth, low birth weight, and stillbirth in the US: a systematic review. *JAMA Netw Open*. 2020;3(6):e208243. doi:10.1001/jamanetworkopen.2020.8243

eTable 1. PRISMA (Preferred Reporting Items for Systematic Reviews and Meta-Analyses) Table and MESH Terms Search

eTable 2. Air Pollution and Preterm Birth

eTable 3. Air Pollution and Low Birth Weight

eTable 4. Air Pollution and Stillbirth

eTable 5. Heat and Preterm Birth

eTable 6. Heat and Low Birth Weight

eTable 7. Heat and Stillbirth

This supplementary material has been provided by the authors to give readers additional information about their work.

© 2020 Bekkar B et al. *JAMA Network Open.*

PRISMA (Preferred Reporting Items for Systematic Reviews and Meta-Analyses) Table

Criteria	Inclusion	Phase I Exclusion	Phase II Exclusion
Population	Specialty-providers, all patients	N/A	N/A
Exposure	Air pollution, particulate matter 2.5 ($PM_{2.5}$), ozone (O_3), heat, temperature, climate change	N/A	N/A
Comparison	Any	N/A	N/A
Primary outcomes	Preterm birth, low birthweight, stillbirth, obstetric outcomes, fertility, infertility, miscarriage, pregnancy loss	Fertility, infertility, miscarriage	Only preterm birth, low birthweight, stillbirth included<br><br>Excluded: pre-eclampsia, asthma if these were only obstetric outcomes
Types of study design	Systematic reviews, RCTs, cohort/comparator study designs, historical studies if comparator is present	Qualitative studies, descriptive studies, studies without comparator, literature reviews	Systematic reviews, historical studies
Setting	United States	United States	United States
Years of publication	2007-2019	2007-2019	2007-2019
Publication type	Published primary studies	Conference proceedings, abstract only, book chapters review	N/A

© 2020 Bekkar B et al. *JAMA Network Open.*

Language	English	English	English

MESH Terms Search

1	premature labor OR premature birth OR low birth weight OR fetus OR infant newborn OR birth weight OR miscarriage OR spontaneous abortion OR habitual abortion OR pregnancy loss OR septic abortion OR legal abortion OR incomplete abortion OR threatened abortion OR missed abortion OR therapeutic abortion OR eugenic abortion OR induced abortion OR congenital OR hereditary OR neonatal diseases OR abnormalities OR congenital abnormalities OR multiple abnormalities OR severe teratoid abnormalities OR cardiovascular abnormalities OR chromosome disorders OR digestive system abnormalities OR eye abnormalities OR lymphatic abnormalities OR musculoskeletal abnormalities OR nervous system malformations OR respiratory system abnormalities OR situs inversus OR skin abnormalities OR stomatognathic system abnormalities OR thyroid dysgenesis OR urogenital abnormalities OR fetal diseases OR erythroblastosis, fetal OR inborn genetic diseases OR congenital hemolytic anemia OR congenital hypoplastic anemia OR inherited blood coagulation disorders OR dwarfism OR hereditary eye diseases OR x-linked genetic diseases OR hemoglobinopathies OR autoinflammatory diseases hereditary OR nervous system heredodegenerative disorders OR inborn errors metabolism OR muscular dystrophies OR hereditary neoplastic syndromes OR genetic skin diseases OR newborn, diseases OR neonatal anemia OR birth injuries OR congenital hyperinsulinism OR neonatal hyperbilirubinemia OR ichthyosis OR infant premature diseases OR severe combined immunodeficiency OR prenatal injuries OR prenatal exposure OR delayed effects OR anomalies	2821326
2	toxic actions OR endocrine disruptors OR environmental pollutants OR aroclors OR coal ash OR pentachlorophenol OR polychlorinated biphenyls OR tetrachlorodibenzodioxin OR tetrachloroethylene OR air pollutants OR soil pollutants OR water pollutants OR hazardous substances OR hazardous waste OR noxae OR alkylating agents OR antimetabolites OR antispermatogenic agents OR carcinogens OR cardiotoxins OR caustics OR cytostatic agents OR cytotoxins OR dermotoxins OR immunotoxins OR irritants OR mutagens OR neurotoxins OR oxidants OR poisons OR pyrogens OR riot control agents, chemical OR teratogens OR 6-aminonicotinamide OR isotretinoin OR thalidomide OR pesticides OR coal OR coal power OR mercury OR sulfur dioxide OR natural gas OR water pollutants OR water pollution OR tracking OR mining OR environmental pollution OR air pollution OR body burden OR environmental exposure OR environmental restoration OR environmental remediation OR food contamination OR waste products	3308621
3	1 AND 2	220513
4	Climate change* OR global warm* OR greenhouse effect* OR temperature extreme*	105820
5	3 AND 4	1096

© 2020 Bekkar B et al. *JAMA Network Open.*

6	Fine particulate OR pm 2.5 OR fine particle*	15354
7	Ozone OR O3	30043
8	6 AND 7	44673
9	5 AND 8	90
10	9 AND English [la]	85
11	Premature labor OR premature birth	44,974
12	10 AND 11	5
13	Low birth weight OR birth weight	107936
14	10 AND 13	6
15	Stillbirth OR still birth OR pregnancy loss	56520
16	10 AND 15	2
17	temperature extreme* OR heat OR global warm* OR greenhouse effect*	318636
18	3 AND 17	2881
19	18 AND English [la]	2752
20	19 AND 11	58
21	19 AND 13	85
22	19 AND 15	75
23	20 or 21 or 22	184
24	19 NOT 23	2697
25	24 AND pregnancy	295

© 2020 Bekkar B et al. *JAMA Network Open.*

eTable 2. Air Pollution and Preterm Birth

KQ1: Is prenatal exposure to air pollution components $PM_{2.5}$ and O_3 associated with increased risk of preterm birth?				
**Reference**	**Study location**	**Study design / N**	**Exposure**	**Outcome: Preterm Birth (PTB) (95% CI)**
Zhu 2018	Georgia	Retrospective cohort<br><br>N= 53,094 births	$PM_{2.5}$ whole-pregnancy; adjusted for infant sex, maternal age, race/ethnicity, education, marital status, prenatal care, smoking, alcohol consumption, season of conception	aOR 1.15 (1.07- 1.25 for exposure to AQI levels > 50 (moderate-hazardous). Black mothers had highest risk but not significantly increased with higher $PM_{2.5}$.<br>Georgia has one of the highest rates of PTB in the US
Basu 2017	California	Retrospective cohort<br><br>N= 231,637 births	$PM_{2.5}$ and 23 constituents, whole-pregnancy; adjusted for maternal age, race, education, infant sex, birth year, apparent temperature, ZCTA-level income, season and date of last menstrual period, geographic region	$PM_{2.5}$-associated ammonium, 21% (17.1-25.4) increased risk; $PM_{2.5}$-associated nitrate, 18% (14.9-21.4); PM $_{2.5}$-assoc bromine, 16% (13.2-21.3) per IQR increase; $PM_{2.5}$ total, 16.4% (13.5-19.5). Inverse associations with chlorine, sodium, sodium ion and vanadium. Greatest risk for Black and Asian mothers, older mothers, some college education, and 32-34 weeks gestation.
Kingsley 2017	Rhode Island	Hospital-based cohort<br><br>N= 61,640 births	$PM_{2.5}$ and black carbon (traffic markers), whole-pregnancy average; adjusted for maternal age, smoking, parity, education, race, insurance, marital status, neighborhood SES	$PM_{2.5}$ aOR 1.04 (0.94-1.15) for modeled levels, 0.86 (0.76-0.98) for monitored levels; black carbon was not associated. Younger, more educated, white population, possibly less exposure.
DeFranco 2016	Ohio	Cohort	$PM_{2.5}$, whole-pregnancy;	$PM_{2.5}$ aOR 1.19 (1.09-1.30) increased risk for highest exposure whole

© 2020 Bekkar B et al. *JAMA Network Open.*

		N= 224,921 singleton births	adjusted for age, race, parity, insurance, education, smoking, birth season and year, infant sex	pregnancy; during 3rd trimester, 1.28 (1.20-1.37). Greatest risk in urban counties especially black and Hispanic populations. Overall, 11% of study population exposed to high $PM_{2.5}$ (15 g/m^3 or higher).
Hao 2016	Georgia	Cohort<br><br>N= 511,658 births	$PM_{2.5}$, O_3, elemental carbon, NO_2, CO, per trimester, whole pregnancy adjusted for maternal education, race, smoking	$PM_{2.5}$ aOR 1.021 (1.006-1.037) per IQR increase whole-pregnancy; 2nd trimester 1.011 (1.001-1.008); O_3 not significant. Higher risk for African-Americans, those with less education.
Johnson 2016	New York City	Cohort/ time series<br><br>N= 258,294 singleton births	$PM_{2.5}$ and NO_2, trimester exposures and whole-pregnancy; adjusted for maternal age, race, education, parity, Medicaid status, BMI, sex of infant and year of conception	No significant association for either pollutant in trimester or whole-pregnancy exposures. Older mothers, non-white mothers, those with less education had higher risk.
Laurent 2016	California	Nested matched case control<br><br>N= 442,314 births	$PM_{2.5}$, ultra-fine PM, NO_2, O_3 whole-pregnancy average; adjusted for maternal age, race, education, income level, BMI, smoking	aOR 1.133 (1.118-1.148) per IQR increase (6.45g/m^3) in $PM_{2.5}$ total, whole-pregnancy; $PM_{2.5}$ constituents strongest for nitrate, ammonium, secondary organic aerosols. O_3 aRR 1.096 (1.085-1.108) per whole-pregnancy IQR increase (11.53 ppb)<br><br>*CA has densest ambient PM measurement network of any state in the U.S.
Mendola 2016	12 U.S. centers	Retrospective cohort	$PM_{2.5}$, PM_{10}, O_3, NO_2, SO_2, CO, weekly and 3 months pre-	$PM_{2.5}$: asthmatics, aOR week 26, 1.52 (1.08-2.15), week 28, 1.43 (1.07-1.92) and week 29,

© 2020 Bekkar B et al. *JAMA Network Open.*

		N= 223,502 births	conception, in pregnant asthmatics; adjusted for maternal age, race, BMI, smoking, alcohol, study site, parity, insurance, marital status and co-morbidities	1.38 (1.10-1.73) per $PM_{2.5}$ IQR increase (5.0 □g/m^3). O_3 negatively associated at 34 weeks per IQR increase (7.9 ppb); risk only shown for non-asthmatics.<br>Risk of PTB higher for Black mothers, public insurance, younger/older, smoked or drank alcohol
Pereira 2016	Rochester, New York	Longitudinal study (Cohort)<br><br>N= 7,121 singleton births	$PM_{2.5}$ average levels by trimester and whole-pregnancy; adjusted for maternal age, parity, exercise, drug use, weight, depression and smoking	aOR 1st trimester, 1.11 (1.04-1.18); 2nd, 1.09 (1.02-1.16); 3rd, 1.06 (1.00-1.13) and whole pregnancy, 1.17 (1.07-1.28) per 1 □g/m^3 increase; only measured to week of birth or 36th week, whichever was earliest. Not significant for premature rupture of membranes
Symanski 2016	Harris County, Texas	Time- series<br><br>N= 222,735 births	$PM_{2.5}$ monthly exposure during pregnancy; adjusted for pollutant levels during the other 4 week periods, season, race, education, age, BMI, support received from infant's father, WIC services, trimester prenatal care initiated, parity, type of payment provided	$PM_{2.5}$ exposure at 1-4 weeks: increased risk of mild (33-36 weeks) PTB, 16% (5-28), moderate (29-32 weeks) PTB, 71% (30-124) and severe (20-28 weeks) PTB, 73% (25-139) PTB per 10 □g/m^3 increase. Significant associations at 9-12, 17-20, 21-24, 25-28, and 29-32 gestational weeks.
Chang 2015	Atlanta metro area	Time-to-event cohort analysis<br><br>N= 175,891 singleton births	$PM_{2.5}$ per trimester, first four weeks, 4 week lag and whole-pregnancy	Significant exposure windows included first 4 weeks, trimesters 1 and 2 and whole-pregnancy; highest RR, trimester 1, 1.07 (1.03-1.11) and whole-pregnancy 1.07 (1.04-1.12). Third

© 2020 Bekkar B et al. *JAMA Network Open.*

				trimester and 4 week lag exposures did not increase risk.
Rappazzo 2015	Pennsylvania, Ohio, New Jersey	Cohort<br><br>N= 1,940,213 singleton births	Fractions of $PM_{2.5}$: elemental carbon, organic carbon, nitrate, sulfate during each week of pregnancy; adjusted for maternal age, race, marital status, education, O_3	Strongest evidence for elemental carbon and sulfate $PM_{2.5}$ fractions. Significant differences found between fractions and at different exposure windows and timing of early delivery. Higher risks for less-educated, unmarried African Americans.
Ha 2014	Florida	Retrospective cohort<br><br>N= 423,719 singleton births	O_3 and $PM_{2.5}$ per trimester and whole pregnancy; adjusted for maternal age, ethnicity, education, marital status, infant gender, prenatal care status, alcohol, smoking, census group income, urbanicity, presence or absence of maternal risk factor, LBW status, co-morbidity	$PM_{2.5}$: significant positive association 2nd trimester and whole-pregnancy; highest 2nd trimester, risk increased 12% (11-14) per IQR increase (2.6 [illegible]g/m^3); for delivery before 32 weeks, positive for 1st, 2nd trimester and whole pregnancy exposures; highest for 2nd trimester, risk increased 22% (18-25). O_3: significant association 2nd trimester and whole-pregnancy, highest for whole-pregnancy, 3% (1-5) per IQR increase (7.1 ppb); for delivery before 32 weeks, positive for 1st, 2nd trimesters and whole-pregnancy, highest for whole-pregnancy, 13% (7-19).
Padula 2014	San Joaquin Valley, California	Retrospective cohort<br><br>N= 263,204 singleton births	$PM_{2.5}$, PM_{10}, CO, NO_2, trimester averages, last month and last 6 weeks of pregnancy; adjusted for maternal age, race, education, prenatal care, birth costs payment	$PM_{2.5}$ whole-pregnancy exposure significantly associated with births from 24-36 weeks, aOR 1.96 (1.68-2.30) between 24-27 weeks; 2nd trimester exposure highest overall at 20-23 weeks, risk increase 2.83 (2.29-3.50). Exposure in the last month of pregnancy positively associated between 20-

				33 weeks, strongest for 20-23 weeks, risk increase 2.84 (2.29-3.52). Higher effects for low SES mothers with second trimester exposure, especially 20-23 weeks, 4.30 (2.85-6.48)
Pereira<br>2014	Connecticut	Longitudinal study of retrospective cohort<br><br>N= 48,208 singleton births	$PM_{2.5}$ per trimester by source in at least two pregnancies delivered vaginally during study period;<br>adjusted for maternal age, parity, smoking	Risk not significantly elevated per IQR increase (2.3 □g/m^3) in whole-pregnancy exposures for $PM_{2.5}$ sources including dust, oil combustion, auto emissions and regional sulfur
Rappazzo<br>2014	Pennsylvania, Ohio, New Jersey	Cohort<br><br>N= 1,940,213 singleton births	$PM_{2.5}$ by week of pregnancy;<br>adjusted for maternal age, race, marital status, education and O_3	4th week of gestation exposure correlated with all periods of premature birth, highest for delivery at 28-31 weeks, aRD 46% (23-69) and 32-34 weeks, aRD 61% (23-100) per 1 □g/m^3 increase, adjusted for O_3. Exposures from birth week and the 2 weeks before birth were also positively associated with all periods of premature birth.<br>Preterm births more likely in non-Hispanic Black women.
Lee<br>2013	Allegheny County, Pennsylvania	Cohort<br><br>N= 34,705 singleton births	$PM_{2.5}$, O_3, PM_{10}, first trimester;<br>adjusted for maternal age, race, parity, smoking, season of birth, year of conception	$PM_{2.5}$, aOR 1.10 (1.01-1.20) increased risk per 4 □g/m^3 increase; O_3 1.23 (1.01-1.50) increased risk per 16.8 ppb increase. Both pollutants associated with PIH and gestational hypertension.
Trasande<br>2013	United States: nationally representative sample	Cross-sectional<br><br>N= 222,359 births	$PM_{2.5}$, O_3, PM_{10}, CO;<br>multi-pollutant model in birth	$PM_{2.5}$ exposure negatively associated (3%/ppm); O_3 mean level associated with $3,632

© 2020 Bekkar B et al. *JAMA Network Open.*

			month at hospital, not home address; adjusted for race, insurance/payment, median income, hospital region, admission month, teaching hospital status, infant sex Note: Smoking, alcohol or drug use not controlled for.	increase in hospital costs per ppb increase
Chang 2012	North Carolina	Time-series<br><br>N= 453,562 births	$PM_{2.5}$ average exposure per trimester and whole-pregnancy using two exposure assessment measures; adjusted for maternal age, race, education, marital status, smoking, birth order, infant sex	Per IQR increase (1.73 □g/m^3) in $PM_{2.5}$, significant associations with 1st, 2nd and whole-pregnancy; highest for whole-pregnancy, 6.8% (.5-13.6). Increases not significant for short-term exposure or 3rd trimester. Higher risk for non-Hispanic Black mothers.
Kloog 2012	Massachusetts	Retrospective cohort<br><br>N= 634,244 singleton births	$PM_{2.5}$, 30 and 90 days before birth and whole-pregnancy; adjusted for maternal age, race, education, income, prenatal care, smoking, infant sex, gestational age, open space near residence, traffic density, mother's health. Used a novel method of exposure prediction at finer spatial resolution.	aOR 1.06 (1.01-1.13) per 10 □g/m^3 in whole-pregnancy exposure; other intervals not significant.
Salihu 2012	Florida	Retrospective cohort<br><br>N= 103,961 singleton births	Median levels of $PM_{2.5}$, PM_{10}, coarse particulates, whole-pregnancy; adjusted for education, race, year of birth, smoking, parity,	No significant association with above-the median exposure to $PM_{2.5}$ overall.<br>aOR 1.08 (1.03-1.13) for PTB and 3-way pollutant interaction.

© 2020 Bekkar B et al. *JAMA Network Open.*

			marital status, prenatal care, sex of infant, anemia, gestational hypertension or diabetes, diabetes, chronic hypertension, preeclampsia, placental abruption or previa, renal disease	Black mothers had greatest morbidity for all particulate exposures and outcomes.
Wilhelm 2011	Los Angeles county, California	Case-control<br><br>N= 241,415 singleton births	Whole-pregnancy average source-specific $PM_{2.5}$, polycyclic aromatic hydrocarbons, O_3, CO, PM_{10}, NO, NO_2, NO_x; adjusted for maternal age, race, parity, education, prenatal care, payment source, mother's birthplace, SES	Positive association with $PM_{2.5}$-associated elemental carbon, organic carbon, biomass burning, ammonium nitrate and diesel burning; highest risk for ammonium nitrate, 21% (16-27) per IQR increase. Population near monitors more likely to be younger, Hispanic, uninsured, less educated, more Medi-Cal or government insurance.
Darrow 2009	Metro-politan Atlanta	Time-series retrospective cohort<br><br>N= 476,489 singleton births	$PM_{2.5}$ total/fractions, PM_{10}, O_3, CO, NO_2, SO_2 from week of delivery, 4 and 6 weeks before; adjusted for maternal race and parity, long-term and seasonal trends, education, gestational week, maternal characteristics	Not significant for $PM_{2.5}$ total, but $PM_{2.5}$ sulfate fraction aRR 1.09 (1.01-1.19) and $PM_{2.5}$ water-soluble metals aRR 1.11 (1.02-1.22) in the week before delivery
Wu 2009	Los Angeles and Orange County, California	Cross-sectional<br><br>N= 81,186 singleton births	Traffic-generated $PM_{2.5}$ and NO_x, whole-pregnancy; adjusted for maternal age, race, prenatal care insurance type, parity, poverty, season of conception, pyelonephritis	Per IQR increase, PTB increased 3% (1-6), 7% (3-12) for less than 35 weeks, and 18% (10-26) for less than 30 weeks per IQR of 1.35 □g/m^3. Highest quartile PM 2.5 exposure, risk increase 81% (71-92) for delivery less than 30 weeks; PIH risk increase 42% (26-59). Rates of premature

© 2020 Bekkar B et al. *JAMA Network Open.*

				birth higher among African American women than other races.

Preterm Birth = delivery at less than 37 weeks completed gestation; some authors provided additional subcategories
$PM_{2.5}$: fine particulate matter
aOR: adjusted odds ratio
AQI: air quality index
ZCTA: zip code tabulation areas
IQR: interquartile range
SES: socio-economic status
LMP: last menstrual period
O_3: ozone
NO_2: nitrous dioxide
CO: carbon monoxide
BMI: body mass index
aRR: adjusted relative risk
SO2: sulfur dioxide
WIC: special supplemental nutrition program for women, infants and children
LBW: low birth weight
PM_{10}: coarse and fine particulate matter
aRD:adjusted risk difference
PIH: pregnancy-induced hypertension
NO: nitrogen oxide
NO_x: nitrogen oxides

© 2020 Bekkar B et al. *JAMA Network Open.*

eTable 3. Air Pollution and Low Birth Weight

**KQ2: Is prenatal exposure to air pollution components $PM_{2.5}$ and O_3 associated with increased risk of low birthweight?**				
**Reference**	**Study location**	**Study design / N**	**Exposure**	**Outcome: Low birthweight (LBW) (95% CI) or as specified**
Nobles 2019	Utah	Retrospective Cohort<br><br>N= 122,203 births	$PM_{2.5}$, O_3, SO_2, NO, NO_2, CO, PM_{10} by trimester, whole-pregnancy and 3 months pre-conception; adjusted for maternal age, race/ethnicity, pre-pregnancy BMI, smoking, alcohol use, parity, insurance type, marital status, history of asthma and ambient temperature	Fetal growth restriction (FGR): $PM_{2.5}$ aRR 1.09 (1.02-1.16) for whole-pregnancy, 1.07 (1.02-1.13) for 3 months pre-conception per IQR increases in exposure (7.39-7.52); not significant for SGA.<br>O_3: negatively associated with FGR and SGA (authors noted an inverse association with ozone and the other pollutants studied). Used physician-diagnosed FGR to improve detection of effect vs. a population standard
Ng 2017	California	Cohort<br><br>N= 1,050,330 singleton births	$PM_{2.5}$ total and five sources, including secondary ammonium sulfate, secondary ammonium nitrate, vehicular emissions, biomass burning and re-suspended soil, by trimester and whole-pregnancy; adjusted for maternal age, race, education, gestational age, year of birth, apparent temperature, neighborhood level % of households below poverty level	Per whole-pregnancy IQR increase: $PM_{2.5}$ total risk increased 4.9% (2.6-7.3), (IQR 6.9 □g/m^3), ammonium sulfate fraction, 7.7% (4.7-10.7), re-suspended soil, 5.6% (3.5-7.7), ammonium nitrate, 3.1% (1.3-4.9).<br>By trimester, effects varied by pollutant; strongest for ammonium sulfate in 1st trimester, 4.1% (2.6-5.7) per IQR increase. Regional differences found between north and south, inland and coastal for several sources.

© 2020 Bekkar B et al. *JAMA Network Open.*

				Lowest risk for college educated and Asian race, highest impact for Black race.
Coker 2016	Los Angeles County, California	Retrospective cohort<br><br>N= 804,726 term singleton births	$PM_{2.5}$, NO, NO_x traffic-related, whole pregnancy; adjusted for maternal age, race, parity, gestational days, and days squared, infant sex, census-level group block ethnic composition and median household income, education, percentage of homes built before 1950	Prevalence 2.4% (2.3-2.5) and 2.6% (2.1-3.2) in areas with highest $PM_{2.5}$/traffic exposures. Note: prevalence for the study population was 2.07%.<br>Highest risk occurred in neighborhoods near roadways and urban core areas, especially for non-Hispanic Black and Asian mothers
Kumar 2016	Chicago, Illinois	Retrospective cohort<br><br>N= 398,120 singleton births	$PM_{2.5}$, PM_{10} per trimester and whole-pregnancy; adjusted for age, marital status, education, neighborhood level of poverty and smoking status	0.97 gm (0.2-1.7) reduction in birth weight per [illegible]g/m^3 increase in $PM_{2.5}$ whole-pregnancy, highest impact in first trimester.<br>Study showed uncertainty with varying distances from monitoring stations. This study area had high exposures generally.
Tu 2016	Georgia	Retrospective cohort<br><br>N= 105,818 term singleton births	$PM_{2.5}$, O_3, whole-pregnancy; adjusted for maternal age, race, education, smoking, prenatal care.<br>Used a novel technique to measure spatial variation of pollutants called Geographically-weighted regression (GWR).	$PM_{2.5}$: positive associations with LBW found only in those urban areas producing higher levels. O_3: significant positive associations only seen in non-urban areas where conditions favored formation of this secondary pollutant.
Twum 2016	Georgia, 9 urban counties	Retrospective cohort	$PM_{2.5}$, average levels, whole-pregnancy; adjusted for	$PM_{2.5}$: aOR 1.36 (1.03-1.79) for exposures in the 75-95%, highest in non-Hispanic black

© 2020 Bekkar B et al. *JAMA Network Open.*

		N= 48,172 term births	maternal age, race, education, marital status, prenatal care, infant sex, smoking and alcohol use	mothers. Rates were not well-correlated with $PM_{2.5}$ levels. Georgia has among the highest rates of low birth weight in the country, and it is increasing;
Coker 2015	Los Angeles County, California	Retrospective cohort<br><br>N= 1,356,304 term singleton births	$PM_{2.5}$, whole-pregnancy; adjusted for maternal age, race, education, parity, gestational length, gestation squared, infant sex	aOR 1.19 (1.02-1.39) increased risk for spatial multi-level modeled exposures of $PM_{2.5}$ per IQR increase (1.96 □g/m^3). Highest impacts in South and Central tracts, possibly due to older homes, lower SES, less access to air conditioning, exposure to other pollutants; highest risk in Black followed by Asian mothers
Ha 2015	Florida	Retrospective cohort<br><br>N= 423,719 singleton births	Residential proximity to power plants, verified by $PM_{2.5}$ measurement; adjusted for maternal age, race, education, marital status, census group income, urban neighborhood	3% (1-4) increased risk for each 5 km shorter distance between home and solid waste plants. These plants conferred higher risk than other lower-$PM_{2.5}$ plants (gas, oil, nuclear). Coal plant proximity correlated with highest adverse birth outcomes; coal plants also emit the most $PM_{2.5}$ along with other pollutants
Hao 2015	United States	Retrospective cohort<br><br>N= 3,389,450 term singleton births	$PM_{2.5}$ by trimester and whole-pregnancy; adjusted for maternal age, race, education, marital status, prenatal care, birth season, infant sex, parity, county-level poverty rate and random effects	Overall, no significant increased risk per 5 □g/m^3 increase of $PM_{2.5}$. Significant risk found in mid-Atlantic region, 1.14 (1.04-1.24), East North Central and West North Central for whole-pregnancy exposures and specific trimesters; negative

© 2020 Bekkar B et al. *JAMA Network Open.*

				association with Mountain region
Lakshmanan<br>2015	Boston, Massachusetts	Cohort<br><br>N= 670 singleton births	Traffic-related $PM_{2.5}$ and black carbon, whole-pregnancy; adjusted for maternal age, race, education, smoking, prenatal stress, season of birth and neighborhood disadvantage z score	$PM_{2.5}$: 0.42 units reduction in BWGA z-score (.06-.79) for males born to obese mothers per IQR increase (1.64 □g/m^3), highest risk group. Suggests maternal status and fetal sex help determine sensitivity to impacts.
Gray<br>2014	North Carolina	Retrospective cohort<br><br>N= 457,642 singleton births	Daily average levels of $PM_{2.5}$ and O_3, whole-pregnancy; adjusted for maternal age, prenatal care, gestational age, marital status, smoking, year of birth, parity, infant sex	$PM_{2.5}$: 3.13 gm (2.93-3.14) reduction in weight per IQR increase (2.3 □g/m^3) at term; O3, 7.4 gm (5.2-9.5) per IQR increase (7.4 ppb). aOR 1.02 (0.99-1.04) for $PM_{2.5}$, 1.06 (1.03-1.09) for O_3. Births studied from 24-42 weeks (not restricted to term).<br>SES level and non-white race correlate with increased exposures and worse outcomes
Ha<br>2014	Florida	Retrospective cohort<br><br>N= 423,719 singleton births	$PM_{2.5}$ and O_3 per trimester; adjusted for maternal age, race, education, marital status, infant sex, prenatal care, alcohol, smoking, season of conception, census group income, urbanicity, maternal risk factor, infection, PTD status, co-morbidity	$PM_{2.5}$ 2nd trimester exposure: 3% (1-6) increased risk per IQR increase (2.6 □g/m^3). O_3 was protective. Results were consistent with multi-pollutant models
Hyder<br>2014	Connecticut Massachusetts	Retrospective cohort	$PM_{2.5}$, measured with land based and satellite exposure	aOR 1.08 (1.04-1.11) per each IQR increase (2.41 □g/m^3) in whole pregnancy exposure,

© 2020 Bekkar B et al. *JAMA Network Open.*

		N= 628,131 singleton births	assessment methods; adjusted for age, race, education, marital status, prenatal care, smoking, type of birth, parity, season of conception, medical risk factors, previous PTD/SGA, gestational age, infant sex	significant for 1 of 2 satellite-based assessment methods. Reduction in birth weight ranged from 6 gm (5-8) to 19 gm (15-23) using the different measurement methods
Laurent 2014	Los Angeles County	Cohort<br><br>N= 960,945 singleton births	$PM_{2.5}$, ultra-fine PM, O_3, NO_2; sources and elements of PM by trimester and whole-pregnancy; adjusted for maternal age, race, education, parity, gestational age, neighborhood income, infant sex	$PM_{2.5}$: aOR 1.02 (1.01-1.31) increased risk per IQR increase (5.82 □g/m^3) whole-pregnancy; O_3 not significant. All sources except shipping associated with modest increases in risk; highest was gasoline. More impact on mothers who were lower-educated, Hispanic, diabetic, chronic hypertension, high BMI.
Savitz 2014	New York City	Cohort<br><br>N= 268,601 singleton births	$PM_{2.5}$ and NO_2 in each trimester and whole-pregnancy; adjusted for age, race, education, parity, gestational age, Medicaid status, year, season and month of conception, neighborhood economic status; smokers excluded	Decreases in weight 1st/2nd/3rd trimester and whole-pregnancy per 10 □g/m^3 increase in $PM_{2.5}$: 18.4 gm/10.5 gm/29.7, 48.4 gm, not significant after 2-pollutant adjustment. Lower birth weights for Black and Asian mothers
Vinikoor-Imler 2014	North Carolina	Cohort<br><br>N= 322,981 singleton births	$PM_{2.5}$, O_3 by trimester; adjusted for maternal age, race, education, marital status, smoking, parity, prenatal care, rural-urban category,	O_3, 3rd trimester aRR 2.03 (1.8-2.3), SGA 1.16 (1.11-1.22) per IQR increase (16.5 ppb); not significant in first or second trimester. $PM_{2.5}$: not significant. Statewide cohort included

© 2020 Bekkar B et al. *JAMA Network Open.*

			month of conception	mothers in both urban and rural areas; Black mothers at highest risk. Single-pollutant and co-pollutant models had similar results.
Basu 2013	California	Cohort<br><br>N= 646,296 term singleton births	$PM_{2.5}$ mass and constituents, whole- pregnancy; adjusted for maternal age, race, education, gestational age, month, season and year of birth, infant sex, apparent temperature exposure, unemployment percentage, home ownership percentage, zip code tabulation area, SES	$PM_{2.5}$ mass (total) associated with 7 gm (4-9) weight reduction per IQR increase (7.56 [illegible]g/m^3); significant associations with several constituents, highest for vanadium, sulfur and iron. Significant association for $PM_{2.5}$ iron only, 6% (2-11) increased risk. Risks greater for younger mothers and varied by Black/Hispanic race/ethnicity
Laurent 2013	Orange County, California	Cohort<br><br>N= 74,416 term singleton births	$PM_{2.5}$, O_3, NO_x, NO_2, CO, PM_{10} using different measurement metrics; adjusted for maternal age, race, parity, insurance status, poverty, gestational age, infant sex	aOR 1.13 (1.02-1.25); 31 gm (26-37) reduction in birth weight, per IQR increase (11.50 ppb) of O_3 whole-pregnancy.<br>$PM_{2.5}$ not significant using monitoring station measurements. No significant increased risk noted for traffic density or proximity to major roads.
Lee 2013	Allegheny County, Pennsylvania	Cohort<br><br>N= 34,705 singleton births	$PM_{2.5}$, O_3, PM_{10} first trimester; adjusted for maternal age, race, parity, smoking, season of birth, year of conception	$PM_{2.5}$ and O_3 per IQR increase not significantly associated with SGA, gestational hypertension and preeclampsia
Trasande 2013	United States	Cross-sectional<br><br>N= 222,359 births	$PM_{2.5}$, O_3, PM_{10}, CO, multi-pollutant model, during birth month at hospital address;	$PM_{2.5}$ associated with increased risk 12% (8-16) per [illegible]g/m^3 increase (single pollutant model); VLBW 8% (5-11) increase.

© 2020 Bekkar B et al. *JAMA Network Open.*

			adjusted for race, insurance/payment, median income, hospital region, admission month, teaching hospital status, infant sex; Note: Smoking, alcohol or drug use not controlled for.	O_3 associated with increased risk VLBW 160% (40-382) per incremental increase above mean levels. Adjusted 3-pollutant model showed 12% (7-16) increase of low birth weight at term per g/m^3 increase $PM_{2.5}$ during birth month
Ebisu 2012	Connecticut, Maryland, Massachusetts, Delaware, New Hampshire, New Jersey, New York, Pennsylvania, Rhode Island, Washing-ton DC, Vermont, Virginia, West Virginia	Retrospective cohort<br><br>N= 1,207,800 term singleton births	$PM_{2.5}$, $PM_{2.5}$ constituents, O_3, PM_{10}, CO, NO_2, SO_2 per trimester and whole-pregnancy; adjusted for maternal age, race, education, marital status, alcohol and smoking, infant sex, gestational age, prenatal care, birth order, delivery method, apparent temperature by trimester, season and year of birth, SES factors	Risk increased 4.9% (3.4-6.5) for $PM_{2.5}$ aluminum, 4.7% (3.2-6.2) for $PM_{2.5}$ elemental carbon 5.7% (2.7-8.8) for $PM_{2.5}$ nickel, 5.0% (3.1-7) for $PM_{2.5}$ titanium per IQR increase whole-pregnancy.<br>Other constituents and pollutants including $PM_{2.5}$ total and O_3 not significant.<br>Highest risk period depended upon specific pollutant exposure. $PM_{2.5}$ elemental carbon and nickel risks higher for white mothers, male infants.
Holstius 2012	Southern California	Time-series<br><br>N= 886,234 term singleton births	Wildfire-associated air pollutants per trimester ((specific concentrations of $PM_{2.5}$ and ozone not measured); adjusted for maternal age, race, education, parity, infant sex, gestational age, infant sex, seasonal effects	1st trimester exposure to wildfire: 3.3 gm (.6-7.2) reduction in birth weight, 2nd trimester, 9.7 gm (4.5-14.8), 3rd trimester, 7.0 gm (2.2-11.8).<br><br>Wildfire-associated PM is more toxic than PM from other sources
Kloog 2012	Massachusetts	Retrospective Cohort	$PM_{2.5}$, 30 and 90 days before birth	Birth weight reduction 8.8 gm (4.4-10.3) for last 30 days, 9.2 gm

© 2020 Bekkar B et al. *JAMA Network Open.*

		N= 634,244 singleton births	and whole-pregnancy; adjusted for maternal age, race, education, infant sex, mean income, prenatal care, gestational age, smoking, percent open space nearby, average traffic density, mother's health	(3.3-15) for last trimester and 13.8 gm (6.1-21.1) for whole-pregnancy exposure, per 10 g/m³ increase in $PM_{2.5}$ after controlling for factors including traffic proximity
Le 2012	Detroit, Michigan	Retrospective cohort<br><br>N= 164,905 singleton births	O_3, PM_{10}, CO, SO_2, NO_2;<br>adjusted for maternal age, race, education, smoking, infant sex, gestational age, prenatal care, birth season, site of residence, long-term exposure trends	SGA at term aOR 1.11 (1.02-1.20) increased risk with highest quartile O_3 exposure in 3rd trimester (>52.75 ppb) during the high season.
Salihu 2012	Florida	Retrospective cohort<br><br>N= 103,961 singleton births	Median levels of $PM_{2.5}$, PM_{10}, coarse particulates, whole-pregnancy; adjusted for education, race, year of birth, smoking, parity, marital status, prenatal care, sex of infant, anemia, gestational hypertension or diabetes, diabetes, chronic hypertension, preeclampsia, placental abruption or previa, renal disease	For $PM_{2.5}$ exposure above the median, aOR 1.07 (1.01-1.12); VLBW 1.14 (1.01-1.29); higher risk for all 3 pollutant classes combined, aOR 1.10 (1.04-1.16) for low birth weight at term.<br>All singleton live births included (not restricted to term).<br>Black mothers had highest odds for all noted morbidity outcomes.
Wilhelm 2011	Los Angeles County, California	Case-control<br><br>N= 220,528 term singleton births	$PM_{2.5}$, PAH, NO, NO_x, NO_2, whole-pregnancy; adjusted for maternal age, race, education, parity, gestational age,	$PM_{2.5}$ (fuel combustion, paved road dust), other traffic pollutants whole-pregnancy 5% increased risk (not significant) per IQR

© 2020 Bekkar B et al. *JAMA Network Open.*

			gestational age squared	increase (0.61-0.83 □g/m^3).<br>Population living near monitors more likely younger, Hispanic, uninsured, on Medi-Cal or government insurance.
Bell<br>2010	Connecticut cut, Massachusetts	Time-series<br><br>N= 76,788 singleton term births	$PM_{2.5}$ mass, constituents and sources, per trimester and whole-pregnancy; adjusted for maternal age, race, parity, marital status, smoking or alcohol, apparent temperature by trimester, infant sex, type of delivery, prenatal care, gestational age	Whole pregnancy: $PM_{2.5}$ zinc, 12% (3-21) increased risk; elemental carbon 13% (3-24); silicon 10% (3-19); aluminum 11 (3-20); vanadium 8% (2-15); nickel 11% (3-19) per IQR increase; $PM_{2.5}$ total not significant. Third trimester: $PM_{2.5}$ zinc associated with 6 gm (1-11) lower weight; elemental carbon 25 gm (3-47); oil combustion 7 gm (1-12); nickel 9 gm (2-15) Constituents vary by source: road, oil burning, traffic-related, salt and regional (sulfur)
Morello-Frosch<br>2010	California	Retrospective cohort<br><br>N= 3,545,177 term singleton births	$PM_{2.5}$, O_3, PM_{10}, CO, NO_2, coarse PM;<br>adjusted for maternal age, race, parity, infant sex, marital status, type of assigned geocode, year and season of birth, neighborhood education, home ownership, poverty and unemployment rates	O_3, 5.7 gm (4.9-6.6) weight reduction per pphm; $PM_{2.5}$ 12.8 gm (11.3-14.3) weight reduction per 10 □g/m^3 increase, whole-pregnancy. Greater risk seen in Black, Hispanic and Asian mothers.
Bell<br>2007	Massachusetts, Connecticut	Retrospective cohort<br><br>N= 358,504 singleton births	$PM_{2.5}$, PM_{10}, CO, NO_2; adjusted for maternal age, race, education, marital status, gestational age, infant sex, smoking, prenatal	$PM_{2.5}$ associated with risk increase of 5.4% (2.2-8.7) per IQR increase (2.2 □g/m^3) for whole-pregnancy exposure; greatest risk during 2nd/3rd trimester.

			care, type of delivery, birth order, weather	Pregnancies 32-43 weeks (not restricted to term).<br>Black mothers appeared to be at greatest risk.<br>Risk of low birth weight also found for CO, SO_2 and PM_{10} exposures.

Low birthweight (LBW) = less than 2500 gm after 37 or more weeks gestation unless otherwise specified
$PM_{2.5}$: fine particulate matter
SO_2: sulfur dioxide
NO: nitrogen oxide (NOx)
NO_2: nitrogen dioxide
CO: carbon monoxide
PM_{10}: coarse and fine particulate matter
BMI: body mass index
aRR: adjusted relative risk
IQR: interquartile range
SGA: weight less than 10% for infant sex and gestational age
aOR: adjusted odds ratio
SES: socio-economic status
BWGA: birth weight for gestational age
PTD: preterm delivery
PM: particulate matter
O_3: ozone
NO_x: nitrogen oxides
VLBW: weight less than 1500 gm
PAH: polycyclic aromatic hydrocarbons
Pphm: parts per hundred million

© 2020 Bekkar B et al. *JAMA Network Open.*

eTable 4. Air Pollution and Stillbirth

KQ3: Is prenatal exposure to air pollution components $PM_{2.5}$ and O_3 associated with increased risk of stillbirth?

Reference	Study location	Study design / N	Exposure	Outcome: Stillbirth*/** (95% CI)
Ebisu 2018	California	Nested case-control<br><br>N= 1,175,116 births; 5,377 stillbirths	$PM_{2.5}$ total and constituents on cause-specific stillbirths; adjusted for maternal age, race/ethnicity, education, food stamp rate, gestational age-adjusted exposure to apparent temperature, natural cubic spline of LMP with 2 degrees of freedom per year	Stillbirths due to fetal growth: aOR 1.23 (1.06-1.44) per IQR (7.23 □g/m^3) increase in $PM_{2.5}$ total; similar for resuspended soil (IQR 0.53) and secondary ammonium sulfate (IQR 2.37). No significant associations found for any pollutant and maternal complication-associated stillbirths.
Mendola, 2017	United States: 12 clinical sites nationwide	Retrospective cohort<br><br>N= 223,375 total singleton births; 992 stillbirths	O_3, IQR increase (difference between 25 and 75%) whole-pregnancy and 1st trimester; asthmatic mothers: $PM_{2.5}$, CO; adjusted for maternal age, race, parity, smoking, alcohol use, insurance, marital status, pre-existing hypertension and diabetes, site, season of conception, birth year, average temperature.	**Whole- pregnancy and first trimester O_3 increased aRR 18-39%; 7-12% per 10 ppb ozone the week before delivery ($p < .05$). Maternal asthma significantly increased risk for whole pregnancy exposure to elevated $PM_{2.5}$
DeFranco, 2015	Ohio	Population-based cohort study<br><br>N= 351,036 total births, 1,848	$PM_{2.5}$ levels by trimester and whole-pregnancy; adjusted for maternal age, race, education level, quantity of	* Increased risk 42% (6-91) for high exposure in third trimester (16.22 □g/m^3), not significant for 1st, 2nd or whole-pregnancy. Mean levels exceeded the US EPA National Ambient Air

© 2020 Bekkar B et al. *JAMA Network Open.*

		stillbirths (after 20 weeks)	prenatal care, smoking, season of conception	Quality Standard during study period.
Green, 2015	California	Retrospective Cohort<br><br>N= 3,012,270 livebirths, 13,999 stillbirths (after 20 weeks)	$PM_{2.5}$, NO_2 whole pregnancy;<br>O_3 3rd Trimester Two- pollutant modeling performed; adjusted for apparent temperature, maternal education, race, age, season of last menstrual period, year of conception, infant sex and air basin of residence	* $PM_{2.5}$ aOR 1.06 (.99-1.13) per 10 □g/m^3 increase, whole-pregnancy. O_3 aOR 1.03 (1.01-1.05) per third trimester 10 ppb increase; for Hispanic mothers, O_3 whole-pregnancy exposure aOR 1.03 (1.01-1.06)
Faiz, 2012	New Jersey	Cohort<br><br>N= 343,077 total births, 980 stillbirths analyzed for $PM_{2.5}$	$PM_{2.5}$, NO_2, SO_2, CO;<br>adjusted for maternal age, race, education level, prenatal care and smoking, time of conception, neighborhood and mean apparent temperature during 1st trimester	* $PM_{2.5}$ not significant. Significant associations with SO_2, NO_2 and CO depending on trimester of exposure

*/**: Stillbirth: fetal death at or beyond 20 weeks (*) or 23 weeks (**)
$PM_{2.5}$= fine particulate matter
LMP: last menstrual period
aOR: adjusted odds ratio
IQR: interquartile range
O_3= ozone
BMI: body mass index
CO= carbon monoxide
aRR; adjusted relative risk
ppb: parts per billion
NO_2= nitrogen dioxide
SO_2=sulfur dioxide

© 2020 Bekkar B et al. *JAMA Network Open.*

eTable 5. Heat and Preterm Birth

KQ4: Is prenatal exposure to heat associated with increased risk of preterm birth?				
**Reference**	**Study location**	**Study design / N**	**Exposure**	**Outcome: Preterm Birth (PTB) (95% CI)**
Avalos 2017	Northern California	Case crossover<br><br>N= 14,466 singleton preterm deliveries	Weekly average apparent temperature week before birth; adjusted for CO, NO_2, SO_2, $PM_{2.5}$, O_3	11.6% (4.1-19.7) increase in overall PTB per 5.6°C increased temp, highest for near-term (36-37 weeks), 22.1% (4.1-44.8), during warm season, also significant for severe (less than 35 weeks) preterm delivery. No confounding by air pollutants
Basu 2017	Northern California	Time-stratified case-crossover<br><br>N=14,466 singleton preterm deliveries (same data as Avalos above)	Weekly average apparent temperature week before birth; adjusted for CO, NO_2, SO_2, $PM_{2.5}$, O_3	11.6% (4.1-19.7) increased risk PTB per 5.6°C increase in temperature. Highest risk for younger mothers, Black, Hispanic, underweight, using Medicaid, smokers, with pre-existing or gestational diabetes or hypertension.
Ha 2017	U.S. 12 clinical sites	Cohort and Case crossover<br><br>N=223,375 singleton births	Extreme ambient heat/cold (greater than 90% or less than 10%), whole pregnancy, by week and week before delivery; adjusted for infant sex, maternal age, race, marital status, parity, BMI, insurance, hypertensive disorders of pregnancy, site, humidity, season of conception, $PM_{2.5}$ and O_3	Extreme heat during weeks 1-7 of pregnancy, aRR of early preterm delivery (less than 34 weeks) 1.11 (1.01-1.21); exposure weeks 15-21, early preterm, 1.18 (1.07-1.29); late preterm (34-36 weeks) 1.18 (1.11-1.27); significant also for exposure during 3 months pre-conception and weeks 8-14. Whole pregnancy heat exposure significant for 34 weeks, aRR 1.21 (1.01-1.44) and 36 weeks, 1.16 (1.06-1.28). 2.8°C heat increase the week before delivery, aOR 1.12-1.16 early delivery risk during warm season only ($p < .05$). Extreme cold during weeks 1-7 was significantly associated with increased

© 2020 Bekkar B et al. *JAMA Network Open.*

				risk for all categories of PTB, but protective for exposures at 8-14 and 22-28 weeks.
Kloog 2015	Massachusetts	Cross-sectional<br><br>N= 473,977 births	Average daily ambient temperature; adjusted for SES, traffic density, $PM_{2.5}$, census tract and mother's current and previous health conditions.<br>Used standard temperature monitoring and a novel method to enhance spatial resolution of average air temperature exposure	Using refined spatial modeling, a 0.26% (-0.28, -0.25) decrease in gestational age and a non-significant increase in PTB was associated with 2.8°C increase whole-pregnancy. Standard monitoring data with same whole pregnancy temperature increase found both an increase in gestational age and higher risk of PTB, (1.02, 1.00-1.05).
Basu 2010	California: 16 counties	Case crossover<br><br>N= 58,681 preterm births	Weekly average apparent temperature at time of delivery; adjusted for CO, NO_2, SO_2, $PM_{2.5}$, O_3	8.6% (6.0-11.3) higher risk of PTB for a 5.6ºC increase during warm season; greatest for younger, Black, or Asian mothers, independent of air pollutants

Preterm Delivery: delivery at less than 37 weeks completed gestation; some authors provided further subcategories as noted in the table
CO: carbon monoxide
NO_2: nitrogen dioxide
SO_2: sulfur dioxide
$PM_{2.5}$: fine particulate matter
O_3: ozone
BMI: body mass index
aRR: adjusted relative risk
aOR: adjusted odds ratio
SES: socio-economic status

© 2020 Bekkar B et al. *JAMA Network Open.*

eTable 6, Heat and Low Birth Weight

KQ5: Is prenatal exposure to heat associated with increased risk of low birthweight?				
**Reference**	**Study location**	**Study design/ N**	**Exposure**	**Outcome: Low birthweight (LBW) (95% C/I)**
Basu 2018	California	Retrospective Cohort<br><br>N= 2,032,601 normal weight and 43,629 tLBW infants	Mean apparent temperature; adjusted for season and year of delivery, maternal age, race, infant sex	13% (4.1-22.7) increased risk of LBW for whole pregnancy exposure, 15.8% (5-27.6) for third trimester, per 5.6°C increase; no confounding by criteria air pollutants but O_3 slight effect modifier.
Ha 2017	United States: 12 clinical sites	Case-crossover<br><br>N= 220,572 singleton births	Ambient temperature, greater than 95% or less than 5% (site specific);<br>adjusted for maternal age, race, marital status, parity, BMI, smoking, alcohol, gestational complications, chronic co-morbidity, insurance, study site, humidity, $PM_{2.5}$, O_3	aRR 2.49 (2.20-2.83) for LBW with whole pregnancy heat exposure, 1.31 (1.15-1.50) for third trimester exposure. Cold exposures significantly associated in 2nd, and 3rd trimesters, as well as whole pregnancy.
Kloog 2015	Massachusetts	Cross-sectional<br><br>N= 453,658 births	Ambient temperature; adjusted for SES, traffic density, $PM_{2.5}$, census tract and mother's current and previous health conditions (lung disease, pregnancy-induced hypertension, gestational and non-gestational diabetes, smoking)	Using enhanced spatial modeling to estimate exposures, term birth weight reduced 16.7 gm per IQR increase (8.4°C) during 3rd trimester; LBW not significantly increased per 2.8°C increase in whole-pregnancy temperature (OR 1.04, CI 0.96-1.13). Trend noted for higher risk in urban areas.

Low birth weight= less than 2500 gm after 37 or more weeks gestation unless otherwise specified
O_3= ozone
BMI: body mass index
$PM_{2.5}$= fine particulate matter

© 2020 Bekkar B et al. *JAMA Network Open.*

aRR: adjusted relative risk
SES: socio-economic status
IQR: interquartile range
OR: odds ratio

© 2020 Bekkar B et al. *JAMA Network Open.*

eTable 7. Heat and Stillbirth

KQ6: Is prenatal exposure to heat associated with increased risk of stillbirth?				
**Reference**	**Study location**	**Study design / N**	**Exposure**	**Outcome: Stillbirth*/** (95% CI)**
Ha, 2017	United States: 12 clinical sites nationwide	Retrospective Cohort, case-crossover<br><br>N= 223,375 total singleton births, 987 stillbirths	Ambient heat or cold, whole-pregnancy, pre-conception and by trimester; adjusted for $PM_{2.5}$ and O_3 exposure, maternal parity, infant sex, BMI, age, race, study site, humidity, season of conception, insurance status	**Whole-pregnancy heat exposure (greater than 90%), aOR 3.71 (3.07-4.47), cold 4.75 (3.95-5.71). Risk increased 6% (3-9) per 1.0°C increase the week before delivery during warm season but not for cold temperatures or other exposure windows. Higher risk for black mothers, extremes of maternal age, uninsured, hypertensive
Basu, 2016	California	Time stratified Case-crossover<br><br>N= 8510 stillbirths (after 20 weeks)	Warm-season mean apparent temperature; adjusted for maternal age, race, education, infant sex	*10.4% higher risk (4.4-16.8) per 5.6°C increase in temperature (average lag 2-6 days), highest risks during weeks 20-25 and 31-33.<br>Greater risk for Hispanics, young mothers, preterm births, less educated

*/**: Stillbirth: fetal death at or beyond 20 weeks (*) or 23 weeks (**)
$PM_{2.5}$= fine particulate matter
LMP: last menstrual period
aOR: adjusted odds ratio
IQR: interquartile range
O_3= ozone
BMI: body mass index
CO= carbon monoxide
aRR; adjusted relative risk
ppb: parts per billion
NO_2= nitrogen dioxide
SO_2=sulfur dioxide

© 2020 Bekkar B et al. *JAMA Network Open.*
